# Psychological distress in Ghana: associations with employment and lost productivity

**DOI:** 10.1186/1752-4458-7-9

**Published:** 2013-03-07

**Authors:** Maureen E Canavan, Heather L Sipsma, Achyuta Adhvaryu, Angela Ofori-Atta, Helen Jack, Christopher Udry, Isaac Osei-Akoto, Elizabeth H Bradley

**Affiliations:** 1 Yale School of Public Health, New Haven, CT, USA; 2 Department of Economics, Yale University, New Haven, CT, USA; 3 Department of Psychiatry, University of Ghana, Legon, Ghana; 4 Institute of Statistical, Social and Economic Research, University of Ghana, Legon, Ghana

**Keywords:** Employment, Mental health, Low-income countries

## Abstract

**Objectives:**

Mental health disorders account for 13% of the global burden of disease, a burden that low-income countries are generally ill-equipped to handle. Research evaluating the association between mental health and employment in low-income countries, particularly in sub-Saharan Africa, is limited. We address this gap by examining the association between employment and psychological distress.

**Methods:**

We analyzed data from the Ghana Socioeconomic Panel Survey using logistic regression (N = 5,391 adults). In multivariable analysis, we estimated the association between employment status and psychological distress, adjusted for covariates. We calculated lost productivity from unemployment and from excess absence from work that respondents reported was because of their feelings of psychological distress.

**Findings:**

Approximately 21% of adults surveyed had moderate or severe psychological distress. Increased psychological distress was associated with increased odds of being unemployed. Men and women with moderate versus mild or no psychological distress had more than twice the odds of being unemployed. The association of severe versus mild or no distress with unemployment differed significantly by sex (P-value for interaction 0.004). Among men, the adjusted OR was 12.4 (95% CI: 7.2, 21.3), whereas the association was much smaller for women (adjusted OR = 3.8, 95% CI: 2.5, 6.0). Extrapolating these figures to the country, the lost productivity associated with moderate or severe distress translates to approximately 7% of the gross domestic product of Ghana.

**Conclusions:**

Psychological distress is strongly associated with unemployment in Ghana. The findings underscore the importance of addressing mental health issues, particularly in low-income countries.

## Background

Globally, nearly 450 million individuals suffer from behavioral or mental disorders [1], accounting for 13% of the global burden of disease [2]. Although addressing the mental health burden has become a global priority, low-income countries are understaffed [3,4] and under-budgeted [4] to deal with this need. Estimates claim that nearly 362,000 mental health workers should be trained to address this need [5]; however, the economic impact of this training for low-income countries is daunting [3,6]. Understanding the link between mental health and lost productivity, as measured by excess unemployment and excess absence from work, may be helpful in assessing the economic value of addressing mental health needs, particularly in low-income countries.

Previous research linking mental health and employment in low-income countries is limited. We know of only a handful of empirical studies from low-income countries on this topic, including those from Uganda [7], Nigeria [8], Ethiopia [9,10], Zimbabwe [11,12], and South Africa [13]. Two studies from Uganda [7] and Nigeria [8] found a strong relationship between mental illness and not having a formal job; however, both samples included a substantial proportion of adolescents and individuals who were not seeking employment in their nonworking populations, which may have biased their results. A study from central Ethiopia [9] showed an increased likelihood of depression associated with employment, but the sample was restricted to rural married women and thus has limited generalizability. Three additional studies reported unadjusted associations between unemployment and mental health disorders; however, these associations were no longer significant after adjusting for other socio-demographic factors [10-12]. In contrast, in a nationally representative sample from South Africa, researchers found a significant association between unemployment and severe psychological distress in multivariable analysis [13]. Given the small number of studies, disparate samples, and inconsistency in results, the association between mental health and employment in low-income countries remains largely unknown.

Accordingly, we sought to examine the association between mental health and employment among adults in Ghana, using the Ghana Socioeconomic Panel Survey, a nationally representative sample of over 5,000 households. We selected Ghana because of the availability of this nationally representative dataset and the country’s recent policy focus on addressing access to and quality of mental health services. We hypothesized that individuals with moderate or severe psychological distress would have higher odds of being unemployed, and if employed, would have excess absence from work compared with individuals with mild or no psychological distress. Our findings can be helpful in understanding the implications of poor mental health for employment, providing potential impetus for policy to address this global burden.

## Methods

### Study design

We conducted a cross-sectional analysis using data from the Ghana Socioeconomic Panel Survey, conducted in 2009–2010 by the Economic Growth Center (EGC) at Yale University and the Institute of Statistical, Social, and Economic Research (ISSER) at the University of Ghana, Legon. This nationally representative survey was designed to monitor living standards and economic conditions in Ghana over time.

The survey employed a two-stage stratified sample design. Enumeration areas (EA) were first randomly selected throughout the 10 regions in Ghana, proportional to population estimates in each region, and then 15 households were selected from each EA. EAs were oversampled in the Upper East and Upper West regions to allow for a sufficient number of households to be interviewed. Overall, 5,009 households were interviewed from 334 EAs, and less than 1% of households refused to be interviewed. Within each household, data were collected pertaining to health, education, demographic characteristics, housing conditions, and farm and non-farm enterprises. Demographic information was collected for all household members. The psychological section was only administered to the head of household, the first spouse, and one additional family member selected at random, all of whom were required to be at least 12 years of age.

### Sample

Interviews were conducted face-to-face with participants. Seventeen teams, each consisting of a supervisor, a senior interviewer, four additional interviewers, and one driver, interviewed a total of 19,167 participants in 5,009 households. We excluded respondents who were younger than 18 years (n = 9,109) and individuals who were out of the workforce including students, homemakers, and disabled individuals (n = 3,706), resulting in a sample of 6,360 adults. From this sample, 969 respondents were excluded due to missing data resulting in a final sample of 5,391 individuals (response rate of 85%).

### Measures

#### Outcomes

Our primary dependent variable was employment status, coded as unemployed or employed. Unemployed individuals included those who reported not working and were either actively seeking work or not seeking work because they thought no work was available.

Employed individuals included those who indicated having at least 1 job outside the home for which they were paid in the last 7 days as well as those who reported not working outside the home because they were engaged in a household farm or non-farm enterprise.

Employed respondents reported the number of days during which they experienced a loss in productivity associated with their feelings of psychological distress over the past 4 weeks. Specifically, questions asked “How many days were you totally unable to work, study or manage your day to day activities because of these feelings?” and “Aside from those days, how many days were you able to work or study or manage your day to day activities, but had to cut down on what you did because of these feelings?” Days where respondents reported having to cut down on what they did were counted as a half day of lost productivity.

#### Primary independent variable

We assessed mental health using the Kessler 10 Psychological Distress Scale (K10), a validated measure of psychological distress [14,15]. The K10 has been used to assess mental health in several countries, has been validated in low-income countries [13], has been shown to be associated with the Composite International Diagnostic Interview (CIDI), and indicates a high probability of meeting criteria for a DSM-IV mental disorder [15]. The K10, a 10-item questionnaire, asks the frequency with which respondents have experienced specific feelings, including tired out, nervous or hopeless, over the past four weeks on a 5-point Likert scale ranging from “none of the time” (scored as 1) to “all of the time” (scored as 5). We summed responses to each item for a total possible range of 10 to 50. For analysis, we created 3 categories based on scores consistent with K10 categories in previous studies [16,17]: 10–24, indicating likely to be well or have mild psychological distress; 25–29 for likely to have moderate psychological distress; and 30–50 for likely to have severe psychological distress.

#### Covariates

Our analysis incorporates several covariates including age, sex, marital status (married, never married, separated/divorced or widowed), education (none, primary or less, middle or secondary and above), region (Western, Central, Greater Accra, Volta, Eastern, Ashanti, Brong Ahafo, Northern, Upper East or Upper West), self-reported health (very healthy, somewhat healthy or unhealthy), religion (Christian, Muslim, traditional or no religion), wealth, and alcohol consumption. Wealth was estimated with a 5-level household asset index constructed using principal component analysis of 47 groupings of durable assets and living conditions (including ownership of a stove, refrigerator, computer or air conditioner as well as if the household uses safe roofing material or electricity for cooking or lighting) (I. Osei-Akoti, personal communication February 12, 2012), [18]. Alcohol consumption was measured using a 4-level categorical response to the question “How many days in the week do you consume alcoholic beverages?” The response categories were none (drank 0 days/week), some (drank 1–4 days/week), high (drank 5–7 days/week), and no response.

### Data analysis

We used standard frequency analysis to describe sample characteristics, the distribution of employment status, and the prevalence of psychological distress. Unadjusted associations between employment status and all independent variables were estimated using chi-square tests and t-tests. We conducted multivariable logistic regression to estimate associations between psychological distress and employment status, adjusted for respondent age, sex, marital status, education, geographic region, religion, wealth, and self-reported health. We tested whether or not sex moderated the relationship between psychological distress and employment status by including the interaction of sex and psychological distress in our logistic regression model. This interaction was significant; thus, we presented models for females and for males separately. We also explored the association between level of psychological distress and place of employment (i.e., employed outside the home versus on a household farm or non-farm enterprise) and found this association to be non-significant for moderate and severe psychological distress (P-values = 0.70 and 0.73, respectively). Analyses accounted for the complex survey design using person-level weighting and household level clustered standard errors and were performed using SAS software, version 9.2 (SAS institute, Cary, NC).

We assessed lost productivity by identifying excess unemployment and excess absence from work for moderate and severe psychological distress compared with mild or no distress. Excess unemployment was calculated as the difference between unemployment rates among individuals with moderate or severe distress and unemployment rates among people with mild or no distress. Excess absence from work, for employed people, was calculated as the difference in the mean number of days individuals reported being unable to work (because of their feelings of distress) between individuals with moderate or severe distress and individuals with mild or no distress.

We calculated the percent of Gross Domestic Product (GDP) represented by this excess unemployment and excess absence from work using an approach previously applied to measure lost productivity due to malaria [19]. We restated the excess unemployment and excess absence from work in full-time equivalent (FTE) units to assess how many FTEs were foregone among those with moderate and severe distress compared with those with mild or no distress. To calculate FTEs represented by excess unemployment, we multiplied the excess percentage of unemployment for moderate and severe distress by the number of individuals in the sample with moderate and severe psychological distress, respectively. To calculate the FTEs represented by excess absence from work, we multiplied the excess proportion of days lost due to moderate and severe psychological distress by the total number of employed individuals within that level of psychological distress. We then summed the FTEs for excess unemployment and excess absence from work. This sum of FTEs for lost productivity was divided by the total number of individuals with moderate or severe psychological distress in our sample to determine the percentage of productive time lost. We then estimated the productive time lost for adults in Ghana with moderate or severe psychological distress by multiplying the percentage of productive time lost for individuals in our sample by the estimated total population of Ghanaian adults with moderate or severe psychological distress. We estimated the percent of potential GDP lost by dividing the number of individuals represented by the productive time lost by the total employed adult population (ages 18 and older) in Ghana. GDP estimations were conducted using Microsoft Excel 2007.

## Results

### Sample characteristics

The overall sample included 5,391 individuals with 13% of respondents classified to have moderate distress, and an additional 8% had severe psychological distress (Table 1). About 91% of respondents were employed. Among the employed adults, 23% were employed outside the home and 77% were employed within the home. Slightly more than half of the sample was female, and the average age was 43 years (standard deviation (SD) 0.24). Nearly three-quarters (73%) of respondents were married; 69% were Christian, and 74% reported themselves to be very healthy. More than one-third of respondents had no education. Approximately 38% of respondents reported drinking no alcohol while only 7% reported drinking most days of the week. A total of 29% were unemployed among participants with severe psychological distress; 15% were unemployed among those with moderate distress, and only 6% were unemployed among those with mild or no distress.

**Table 1:** Description of Ghanaian adults (18 and over) by psychological distress and socioeconomic characteristics (N = 5,391)

	Overall *	**Employed**^**§**^	**Unemployed seeking work**^**§**^	
	**N = 5,391**	**N = 4,855 (91.1%)**	**N = 536 (8.9%)**	**P-value**^**a**^
Age	42.5 (0.24)	42.9 (0.26)	38.7 (0.82)	<0.001
Sex				0.580
Female	2897	90.9%	9.1%	
Male	2494	91.3%	8.7%	
Marital Status				<0.001
Married	3945	91.6%	8.4%	
Separated/Divorced	470	93.4%	6.6%	
Widowed	355	94.7%	5.3%	
Never Married	621	83.0%	17.0%	
Education				<0.001
None	1875	85.7%	14.3%	
Primary or Less	796	93.5%	6.5%	
Middle	1981	94.1%	5.9%	
Secondary and Above	739	93.6%	6.4%	
Region				<0.001
Western	562	83.6%	16.4%	
Central	540	96.0%	4.0%	
Greater Accra	388	94.6%	5.4%	
Volta	672	96.6%	3.4%	
Eastern	668	96.7%	3.3%	
Ashanti	869	95.7%	4.3%	
Brong Ahafo	454	95.5%	4.5%	
Northern	850	81.8%	18.2%	
Upper East	251	60.2%	39.8%	
Upper West	137	87.0%	13.0%	
General Health				0.238
Very Healthy	4003	91.4%	8.6%	
Somewhat Healthy	987	91.1%	8.9%	
Unhealthy	401	87.8%	12.2%	
Religion				<0.001
Christian	3656	93.0%	7.0%	
Muslim	848	87.3%	12.7%	
Traditional	544	83.0%	17.0%	
No Religion	343	90.7%	9.3%	
Wealth Quintiles				<0.001
Lowest 20%	951	79.1%	20.9%	
21–40%	1225	85.5%	14.5%	
41–60%	1047	94.1%	5.9%	
61–80%	1052	97.4%	2.6%	
Highest 20%	1116	97.7%	2.3%	
Alcohol Consumption				0.315
None (0 days/week)	2,023	90.2%	9.8%	
Some (1–4 days/week)	905	90.8%	9.2%	
High (5–7 days/week)	403	93.5%	6.5%	
No Response	2,032	91.7%	8.3%	
Psychological Distress				<0.001
Mild/No Distress	4274	93.8%	6.2%	
Moderate	701	84.6%	15.4%	
Severe	416	71.3%	28.7%	

*Overall values are reported in unweighted Ns.

^§^Values are reported in weighted row percent for categorical variables and weighted mean (standard error) for continuous variables.

^a^P-values are given for weighted chi-square test for categorical variables and *T*-test for continuous variables.

### Association between employment status and psychological distress

In unadjusted analysis, men and women with moderate psychological distress had more than twice the odds of being unemployed (for men, odds ratio (OR) = 2.5, 95% confidence interval (CI): 1.5, 4.0; for women, OR = 2.9, CI: 2.0, 4.0) compared with men and women with mild or no psychological distress. The interaction between sex and severe psychological distress was significant (P-value for interaction <0.001); men with severe psychological distress had over 10 times the odds of being unemployed (OR = 10.8, CI: 6.8, 17.0) compared with men with mild or no distress, whereas the odds of women with severe distress being unemployed was 3.9 (CI: 2.6, 6.0) times the odds of women with mild or no distress.

In adjusted analysis for both men and women, those with moderate versus mild or no psychological distress had more than twice the odds of being unemployed (for men, adjusted OR = 2.0, CI: 1.2, 3.5; for women, adjusted OR = 2.1, CI: 1.5, 3.1) (Table 2). This adjusted association of severe distress compared with mild or no distress with unemployment differed significantly by sex (P-value for interaction=0.004). Among men, the adjusted OR was 12.4 (CI: 7.2, 21.3) whereas the association was much smaller among women (adjusted OR = 3.8, CI: 2.5, 6.0). Regression diagnostics indicated a high level of correlation between alcohol consumption and wealth. In order to improve overall model fit, we retained wealth in the multivariable model and excluded alcohol consumption.

**Table 2:** **Multivariate logistic regression model assessing working status among Ghanaian female and male adults**^**¥**^

	**Females**	**Males**
	**(N = 2,897)**	**(N = 2,494)**
	**Unemployed**^**§**^	**Unemployed**^**§**^
	**Adjusted OR (95% CI)**	**Adjusted OR (95% CI)**
Age	0.97 (0.95,0.98)*	0.99 (0.98, 1.01)
Marital Status		
Married	0.46 (0.25, 0.84)*	0.20 (0.11, 0.35)*
Separated/Divorced	0.61 (0.26, 1.45)	0.30 (0.10, 0.89)*
Widowed	0.44 (0.13, 1.47)	--
Never Married	Reference	Reference
Education		
None	2.29 (1.13, 4.66)*	2.08 (1.07, 4.03)*
Primary or Less	1.60 (0.75, 3.44)	1.20 (0.57, 2.52)
Middle	1.86 (0.93, 3.74)	0.94 (0.49, 1.82)
Secondary and Above	Reference	Reference
Region		
Western	1.60 (0.546, 4.78)	1.49 (0.45, 4.91)
Central	0.45 (0.13, 1.52)	0.23 (0.07, 0.82)*
Greater Accra	Reference	Reference
Volta	3.52 (1.02, 12.15)*	0.28 (0.06, 1.27)
Eastern	2.71 (0.87, 8.45)	0.34 (0.07, 1.70)
Ashanti	2.48 (0.79, 7.74)	0.74 (0.14, 3.79)
Brong Ahafo	1.42 (0.37, 5.42)	0.77 (0.13, 4.74)
Northern	2.75 (0.91, 8.32)	0.46 (0.15, 1.46)
Upper East	13.78 (4.398, 43.27)*	2.53 (0.68, 9.41)
Upper West	3.27 (0.88, 12.17)	0.88 (0.19, 3.97)
General Health		
Very Healthy	Reference	Reference
Somewhat Healthy	1.09 (0.698, 1.71)	1.10 (0.55, 2.20)
Unhealthy	1.83 (1.00, 3.35)*	1.23 (0.55, 2.75)
Religion		
Christian	Reference	Reference
Muslim	0.69 (0.41, 1.16)	0.80 (0.44, 1.46)
No Religion	1.44 (0.75, 2.79)	0.90 (0.43, 1.87)
Traditional	0.84 (0.52, 1.36)	1.17 (0.65, 2.11)
Wealth Quintiles		
Lowest 20%	7.49 (3.66, 15.35)*	11.96 (5.16, 27.72)*
21–40%	9.39 (4.58, 19.24)*	6.78 (2.77, 16.47)*
41–60%	2.41 (1.07, 5.40)*	3.54 (1.00, 12.53)*
61–80%	0.67 (0.28, 1.62)	1.50 (0.54, 4.18)
Highest 20%	Reference	Reference
Psychological Distress		
mild/No Distress	Reference	Reference
Moderate	2.14 (1.46, 3.14)*	2.02 (1.17, 3.49)*
Severe	3.84 (2.47, 5.97)*	12.41 (7.23, 21.28)*

^§^ Reference level of logistic model: Employed individual.

¥ p-value for interaction between sex and severe psychological distress: 0.004.

*Indicates that OR is significant at 0.05 level.

### Lost productivity and psychological distress

Lost productivity was derived from excess unemployment and excess absence from work. Excess unemployment among individuals with moderate or severe psychological distress was 11.1% and 24.4%, (Table 3) respectively (derived from 17.7% and 31.0% unemployment among people with moderate and severe distress, respectively, compared with 6.6% unemployment among people who had mild or no psychological distress). This excess unemployment was equivalent to 78 and 101 individuals with moderate and severe distress, respectively.

**Table 3:** Distribution in excess days of unemployment by psychological distress level among Ghanaian adults (N = 5,391)

	**n**	**Number unemployed**	**Percent unemployed**	**Excess percent unemployed**	FTE*
Mild/No Psychological Distress	4,274	283	6.6%	Reference	**--**
Moderate Psychological Distress	701	124	17.7%	11.1%	**78**
Severe Psychological Distress	416	129	31.0%	24.4%	**101**
Total Excess Unemployment (FTE)					**179**

*Individual full-time work equivalent units (FTE) correspond to excess percentage multiplied by n for each level of psychological distress.

Excess absence from work among individuals with moderate and severe psychological distress was 2.9% and 7.8%, respectively; this was derived from 2.2 days per month (or 7.9% of a 28-day month) of impeded work and 3.6 days per month (or 12.8% of a 28-day month) of impeded work among people with moderate and severe distress, respectively (Table 4). The excess absence from work was equivalent to approximately 18 and 23 employed individuals with moderate and severe distress, respectively.

**Table 4:** Distribution in excess absence from work by psychological distress level among employed Ghanaian adults (N = 3,103)

	**n**	**Days with absence from work**^**a**^	**Days with partial absence from work**^**b**^	**Total days affected**^**c**^	**Percent absence from work**^**d**^	**Excess percent absence from work**	FTE*
Mild/No Psychological Distress	2,463	0.6 (0.05)	1.6 (0.14)	1.4 (0.11)	5.0%	Reference	**--**
Moderate Psychological Distress	414	1.1 (0.10)	2.4 (0.32)	2.2 (0.25)	7.9%	2.9%	**18**
Severe Psychological Distress	226	2.1 (0.26)	3.1 (0.43)	3.6 (0.36)	12.8%	7.8%	**23**
Total Excess Absence from Work (FTE)							**41**

*Individual full-time work equivalent units (FTE) correspond to excess percentage multiplied by n for each level of psychological distress.

^a^Mean days (SE) individuals completely unable to work over the past 28 days.

^b^Mean days (SE) individuals forced to work reduced amount beyond the days they were completely unable to work over the past 28 days.

^c^Mean days (SE) individuals completely unable to work and days individuals forced to work a reduced amount over the past 28 days with days of excess absence from work each accounting for a half day of work; all values are capped at 28 days.

^d^Percentage of total days affected over the past 28 days.

Summing these FTEs, the lost productivity is equivalent to 96 individuals with moderate distress and 124 individuals with severe distress, or 220 total individuals. Extrapolating our results to the country of Ghana, which has approximately 13,446,427 adults, we estimated that 20.7% or 2,783,410 individuals may be affected by moderate or severe psychological distress. Lost productivity may therefore represent 19.7% of this group, or 548,732 individuals. These individuals represent approximately 6.8% of the adult working population of 8,067,856 Ghanaian adults. Therefore, we estimate that lost work among people with psychological distress may be represented by approximately 6.8% of the GDP.

## Discussion

People with elevated psychological distress had much higher odds of being unemployed compared with people with mild or no psychological distress. This effect of psychological distress on unemployment was significantly larger for men than for women. The lost productivity associated with moderate and severe psychological distress represented a loss of nearly 7% of GDP, far higher than estimated GDP reportedly lost due to malaria [20].

Our study represents one of the first analysis of this relationship between mental health and employment using a nationally representative sample of adult men and women in a low-income country. Additionally, our analysis examines severe psychological distress but also identifies an association between unemployment and moderate psychological distress, both of which have been shown to be associated with negative health outcomes in previous research in high-income countries [21-23]. The results from this study demonstrate the importance of addressing mental health in the adult population. Previous literature has shown positive results when high-income countries prioritize mental health. Beginning in 2007, the United Kingdom began a large scale financial investment in improving access and treatment for mental health, specifically addressing depression and anxiety [24]. In pilot data from two locations, Doncaster and Newham, the increased investment showed a significant reduction in the number of clinical cases of depression and anxiety as well as a significant increase in the number of patients who returned to work [24]. Additionally, after 2 years of prioritized national focus on mental health, the program has nearly met targets for number of individuals seen and for recovery rates and has exceeded targeted numbers for moving individuals off of sick pay and state benefits [25].

At present, the country of Ghana has shown a commitment to addressing the mental health burden with its recent passage of the Mental Health Bill, and thus may serve as an example for other low-income countries in the region. Legislation appropriating finances and other resources is a first step for ensuring better care among people suffering from poor mental health and for reducing its associated stigma. Our analysis suggests that there is a need for investment of resources to address lost productivity associated with mental health.

Despite the strong statistical association between psychological distress and unemployment, causality cannot be established as the analysis is based on cross-sectional data. Studies assessing mental health and employment in high-income countries [26-30] demonstrate that causality is complex. An inability to find work may result in higher levels of psychological distress or, conversely, those with higher levels of psychological distress may be less able to find work; in some cases, both causal paths may be present. Additionally, the direction of the association between employment and psychological distress may not be unilateral. Higher levels of psychological distress among an employed population have been observed due to difficult working conditions [31,32] or problems associated with underemployment [29], thus we could have underestimated the association between unemployment and psychological distress. Although it is important not to infer causality from our findings, this is nonetheless one of the first large-scale studies establishing a robust association between psychological distress and employment in sub-Saharan Africa. Additionally, the magnitudes of the estimated associations are large, and should serve as motivation for prospective studies to evaluate the gains in productivity that might be achieved when mental illness is adequately managed or treated.

Our findings should be interpreted in light of additional limitations. First, the survey did not measure average number of hours worked per day. If this is lower for people with moderate or severe psychological distress, then we may have underestimated employment associations. Second, estimates of GDP can vary based on assumptions made during its computation. We calculated the percent of GDP represented by the lost productivity among people with moderate and severe psychological distress with methods that have been commonly used to study the indirect financial costs associated with disease [19,33-35], and we applied a conservative estimate of all working age individuals as the population denominator. Although exact numbers vary based on how GDP association is calculated [19], WHO estimates show that the malaria accounts for less than 4% of the overall GDP in Ghana [20]. Highlighting the GDP cost of lost productivity associated with psychological distress can be useful for prioritizing mental health spending over other illnesses. It is important to note that our estimates do not provide casual evidence; however, the magnitudes of the estimated associations are large and should motivate prospective cohort studies and randomized controlled trials to rigorously evaluate the gains in productivity that might be achieved by addressing mental health and other barrier to full employment. Because our estimates depend on extrapolations to the full population, they are speculative, not definitive, and additional studies to replicate these findings would be helpful. Last, our response rate was 85%, and respondents differed significantly from non-respondents in marital status, education, region, religion and wealth, giving rise to potential for response rate bias; however, we did adjust for these variables in the multivariate analysis to mitigate this concern.

## Conclusion

In summary, poor mental health accounts for a substantial portion of the global burden of disease [2], but the treatment rates in low- and middle-income countries are low [36]. We found a strong association between psychological distress and unemployment, and among those working, psychological distress accounted for substantial amount of lost productivity. These findings underscore the importance of addressing mental health issues, particularly in low-income countries, where employment is critical for economic growth.

## Competing interests

The author(s) declare that they have no competing interests.

## Authors’ contributions

MEC participated in the conception and design of the study, carried out the statistical analysis, and drafted the manuscript. HLS helped with interpretation of data and drafting the manuscript. AA helped with interpretation of data and revising of the manuscript. AOA helped to draft the manuscript. HJ helped to draft the manuscript. CU helped with design and acquisition of data and revising of the manuscript. IOA helped with design and acquisition of data. EHB helped with conception of the study, participated in its design and coordination, aided in interpretation of the data and helped draft the manuscript. All authors read and approved the final manuscript.
